# Characterization of an Alkali- and Halide-Resistant Laccase Expressed in *E. coli*: CotA from *Bacillus clausii*

**DOI:** 10.1371/journal.pone.0099402

**Published:** 2014-06-10

**Authors:** Søren Brander, Jørn D. Mikkelsen, Kasper P. Kepp

**Affiliations:** 1 Technical University of Denmark, DTU Chemical Engineering, Kongens Lyngby, Denmark; 2 Technical University of Denmark, DTU Chemistry, Kongens Lyngby, Denmark; Center for Nanosciences and Nanotechnology, Mexico

## Abstract

The limitations of fungal laccases at higher pH and salt concentrations have intensified the search for new extremophilic bacterial laccases. We report the cloning, expression, and characterization of the bacterial cotA from *Bacillus clausii*, a supposed alkalophilic ortholog of cotA from *B. subtilis*. Both laccases were expressed in *E. coli* strain BL21(DE3) and characterized fully in parallel for strict benchmarking. We report activity on ABTS, SGZ, DMP, caffeic acid, promazine, phenyl hydrazine, tannic acid, and bilirubin at variable pH. Whereas ABTS, promazine, and phenyl hydrazine activities vs. pH were similar, the activity of *B. clausii* cotA was shifted upwards by ∼0.5–2 pH units for the simple phenolic substrates DMP, SGZ, and caffeic acid. This shift is not due to substrate affinity (K_M_) but to pH dependence of catalytic turnover: The k_cat_ of *B. clausii* cotA was 1 s^−1^ at pH 6 and 5 s^−1^ at pH 8 in contrast to 6 s^−1^ at pH 6 and 2 s^−1^ at pH 8 for of *B. subtilis* cotA. Overall, k_cat_/K_M_ was 10-fold higher for *B. subtilis* cotA at pH_opt_. While both proteins were heat activated, activation increased with pH and was larger in cotA from *B. clausii*. NaCl inhibited activity at acidic pH, but not up to 500–700 mM NaCl in alkaline pH, a further advantage of the alkali regime in laccase applications. The *B. clausii* cotA had ∼20 minutes half-life at 80°C, less than the ∼50 minutes at 80°C for cotA from *B. subtilis*. While cotA from *B. subtilis* had optimal stability at pH∼8, the cotA from *B. clausii* displayed higher combined salt- and alkali-resistance. This resistance is possibly caused by two substitutions (S427Q and V110E) that could repel anions to reduce anion-copper interactions at the expense of catalytic proficiency, a trade-off of potential relevance to laccase optimization.

## Introduction

Laccases (EC 1.10.3.2, *p*-diphenol dioxygen oxidoreductases) belong to the class of multi-copper oxidases, characterized by four redox-active copper ions organized into three spectrally distinct sites (T1, T2, and T3). Laccases catalyze the one-electron oxidation of four equivalents of a reducing substrate, and in turn reduce one dioxygen molecule completely to water [1], [2]. They oxidize a wide range of substrates, and this power can be further enhanced by the use of mediators such as 2,2'-azino-bis(3-ethylbenzothiazoline-6-sulphonic acid) (ABTS). These properties, along with their substantial stability, have made laccases widely explored as biocatalysts in diverse industrial applications such as delignification, stabilization of beverages, degradation of chlorophenols and dyes, in fine biochemicals and pharmaceutical industries (green organic chemistry), biosensors, and fuel cells [3]–[6].

A major challenge of laccase engineering is that the catalytic proficiency and stability of enzymes is dramatically dependent on environmental variables such as pH, temperature, ionic strength, and co-solvents. Industrial processes are often best employed at non-natural conditions, e.g. stirring conditions or extreme pH, temperature, or ionic strength. Fungal laccases are often active at acidic pH and low ionic strengths, where the proteins are much less stable [7]. Thus, recent efforts have been directed both towards identifying new alkalophilic and halophilic laccases [8]–[12], and towards improving the alkalophilicity of fungal laccases by e.g. directed evolution [7].

While most investigated laccases are of fungal origin, the last decade has seen a range of bacterial laccases being reported [13], [14]. The first characterized bacterial laccase, the spore coat protein A (CotA) from *Bacillus subtilis* [15] displayed high thermostability and a middle-range redox potential. The laccase has later been shown to oxidize a large variety of substrates, such as substituted aromatic compounds [16] and a number of important dyes [17]. In addition, it can function as a bilirubin oxidase [18], a low-affinity nitrous oxide reductase [19], and a manganese oxidase [20].

These capabilities of *Bacillus subtilis* cotA, combined with its impressive stability and the well-established bacterial expression protocols, have initiated a wealth of studies on cotA proteins. The orthologs from *Bacillus pumilus* [21], *Bacillus halodurans* [22], *Bacillus sp. HR03* [23], and *Bacillus licheniformis* [24] have been expressed heterologously in *E. coli*, and those of *Bacillus sp. ADR* [25], *Bacillus amyloliquefaciens LC02* [26], *Bacillus SF* [27], [28], *Bacillus sp. VUS* [29], *Bacillus sp. XJT-7* [30], *Bacillus vallismortis* [31], and *Bacillus thuringiensis* [32] have been expressed from the native host.

A general tendency of the bacterial laccases in comparison to fungal laccases is higher stability at variable pH and temperature, however usually at the cost of having reduced redox potentials than their fungal orthologs [13], an interesting example of functional trade-offs that are often a challenge to protein engineering. Whereas fungal laccases are strongly inhibited by hydroxide ions [33], bacterial laccases are known to often have higher optimal pH-values (pH_opt_) of catalysis. For example, pH_opt_ for *Trametes versicolor* laccase oxidation of 2,6-dimethoxy phenol (DMP) is 3.4 [34] while it is 7.0 for *Bacillus subtilis* cotA [35].

Laccases could be used in a number of future applications where neutral or alkaline pH is desirable, including e.g. hair dying, in alkaline organic synthesis reactions, in laundry detergents, for oxidation of natural and synthetic dyes, bioremediation at neutral pH, and for direct oxidation of phenol-containing biopolymers, with phenolic substrates typically having pK_a_ values near 9–10 [36], [37].

This paper reports the cloning, expression in an *E. coli* strain BL21(DE3), and characterization of a previously uncharacterized cotA from the gram-positive *Bacillus clausii* KSM-K16. Showing optimal growth near pH 9 [38], this species is known to produce alkali-, heat-, and detergent-resistant proteases such as the laundry M-protease with optimal enzymatic activity at pH 12.3 [39]. Since expression and handling protocols affect protein activation and consequently observed activities, as shown in this paper, to enable a strict benchmark for characterization, we simultaneously characterized the cotA from *Bacillus subtilis* 168 after expressing it in the same *E. coli* strain.

We found that the cotA from *B. clausii* has activity profiles shifted towards higher pH compared to *B. subtilis* cotA, notably for DMP, SGZ, and caffeic acid. This finding is in accordance with the optimal growth conditions of *B. clausii*. Both enzymes displayed substantial heat activation, as previously reported for *B. subtilis* spores [15], but for *B. clausii* cotA, the activation was much larger at higher pH. At pH 8, both enzymes displayed substantial activation in NaCl before inhibition became dominant. However, at lower pH, strict inhibition was observed as seen previously for fungal laccases usually assayed at low pH. The new cotA is more resistant to alkaline and high salt conditions, but at the expense of a∼10 fold loss of catalytic proficiency, as estimated from k_cat_/K_M_, providing a new example of trade-off in laccases.

## Materials and Methods

### Cloning protocol

The gene of *B. clausii* cotA (see sequence alignment in Figure S1 in File S1) was identified using protein BLAST with *B. subtilis* cotA as template against the genomic sequence of *B. clausii* KSM-K16. The codon-optimized gene was synthesized with restriction sites NcoI and KpnI by GeneArt Invitrogen in a pMK vector, and amplified by PCR with primers pMK-f 5′-GTG CTG CAA GGC GAT TAA GT-3′ and pMK-r 5′-GAG TCA GTG AGC GAG GAA GC-3′. The 1806 bp PCR product was digested with NcoI HF and KpnI HF (Fermentas). The vector pETM13 (kind gift of Dr. Günther Stier, Heidelberg University) was digested with NcoI and KpnI (Fermentas), treated with shrimp alkaline phosphatase (Fermentas) and resolved in a 1% agarose gel with 0.1% crystal violet (Sigma-Aldrich). A band from the linearized vector was identified on a white light table and subsequently cut out.

The gene and linearized vector were purified with Illustra GFX PCR DNA and Gel Band Purification Kit (Illustra) and ligated with a T4 ligase (Fermentas) to yield the *Bacillus clausii* cotA expression vector p133. The cotA gene from *Bacillus subtilis* 168 was prepared by PCR amplification from genomic DNA using the primers 5'-ATC GTC TCT CAT GAC ACT TGA AAA ATT TG-3' and 5'-GTC GGT ACC TTA TTT ATG GGG ATC AG-5' to yield a 1542-bp PCR product. The gene was digested in two steps with BsmBI (New England Biolabs) and KpnI HF (Fermentas) which allowed for cloning into the previously prepared pETM13 to produce the *Bacillus subtilis* expression vector p130. P130 and P133 were propagated in DH5α, confirmed by DNA sequencing, and sub-cloned into BL21(DE3), allowing IPTG-induced expression of cotA proteins with no fusions or tailing amino acids.

### Expression protocol

Protein expression was carried out in semi-anaerobic conditions [40], which have been shown to enhance copper loading into cotA type laccases. The expression strains were grown in LB medium to OD 0.6 at 180 rpm and 30°C followed by induction to 100 µM IPTG and 250 µM CuCl_2_. Incubation was extended at 120 rpm and temperature 25°C for four hours followed by 16 hours of static incubation at 25°C.

After the static incubation cells were harvested by centrifugation for 20 minutes at 12000 g and re-suspended in 20 mL cold lysis buffer 50 mM MOPS pH 7.8, 200 mM NaCl, 5% glycerol, 0.5 mM Pefabloc SC (Fluka), 0.01% Lysozyme. Cells were sonicated for five minutes on ice (UP400S with microtip H7, Hielscher) in pulses of 30 seconds at maximum power.

The cell lysate was incubated in a water bath for 30 minutes at 70°C, which improved cell lysis and precipitated thermo sensitive proteins. The protein solution was clarified by twice decanting the supernatant after centrifugation 30 minutes at 12000 g and finally filtered through a 0.45 µm cellulose filter. The protein was concentrated on a Vivaspin20 with 30 kDa cutoff (Sartorius), and the buffer was changed to 20 mM MOPS pH 7.6, 50 mM NaCl, and purified on a source Q15 anion exchange column with a linear gradient between 50 mM and 1 M NaCl in MOPS pH 7.6 on an ÄKTA purifier. Fractions were collected based on the absorption at 280 and 600 nm and buffer was changed into 20 mM MOPS pH 7.8 during the final concentration of the samples, using the Vivaspin20 system (for an SDS page gel, see Figure S2 in File S1).

### Activity assays

Optimal pH for catalytic activity was determined for eight diverse substrates by measurement of product formation rates in 50 mM McIlvaine buffer with pH range 2.6–8.0, MOPS with pH-range 7.25–8.6, and CHES with range 8.6–10. All measurements were performed on a Tecan Pro Infinite 200 multiplate reader at 27°C by monitoring the kinetic trace for five minutes in order to calculate the initial rate by linear regression. The eight substrates and absorption wavelengths were 200 µM ABTS, 420 nm; 1 mM 2,6-dimethoxyphenol (DMP), 468 nm; 50 µM syringaldazine, 530 nm; 1 mM caffeic acid, 485 nm; 1 mM promazine, 520 nm; 200 µM tannic acid, 355 nm, 1 mM phenylhydrazine, 400 nm, and 0.0025% bilirubin mixed isomers, 440 nm.

All measurements were done in triplicates along with duplicate measurements of auto-oxidation with milliQ-water added instead of enzyme. ABTS and SGZ exhibited no auto-oxidation in the investigated pH range, whereas DMP, caffeic acid, and tannic acid showed steeply increasing auto-oxidation above pH ∼8.5, and phenylhydrazine turned yellow below pH 4 which complicated the readings. Promazine displayed substantial auto-oxidation across the entire pH range. Bilirubin oxidase activity was measured as the depletion of substrate rather than the formation of product. Despite using substrate concentrations relevant to the full range of the spectrometer, the reaction was not linear for all pH. The reported pH activity profiles are shown after subtraction of the auto-oxidation rates (full data in Figure S3 in File S1). Furthermore, activity also depended to some extent on the buffer, affecting the activity curves across pH ranges where several buffers were used. In order to fully understand this effect, activities were measured at overlapping pH values using both relevant buffers, enabling us to quantify the buffer effects and correct for these. The raw data are shown in Figure S3 in File S1, whereas the activities reported in the main paper were corrected for the buffer effect.

### Stability estimation

The protein stability was investigated at 50°C and 70°C for the *B. clausii* and *B. subtilis* laccases in parallel, by incubating both enzymes at these temperatures and at pH 8, 9, or 10 for variable amounts of time. 200 µL laccase solutions in 20 mM buffer was incubated in a 1.5 mL centrifuge tube on a temperature calibrated Eppendorf themomixer, and at different time points, 10 µL was retracted and immediately placed in a chilled 96-plate well with 90 µL milliQ water. The activity was measured in a new 96-well plate by adding 180 µL 500 µM ABTS in 200 mM McIlvaine buffer pH 5 to 20 µL chilled protein sample and measuring the initial rate at 420 nm. A secondary thermal stability experiment was carried out at temperatures 50°C, 70°C, and 80°C with samples being retracted during a 25-hour session and immediately assayed on a Perkin Elmer Lambda 20 spectrophotometer fitted with a temperature controlled cuvette changer. Additional residual activity curves for estimating optimal pH were produced at 5 hours incubation at 50°C are shown in Figure S4 in File S1, and after 20 hour incubation at 30°C in Figure S5 in File S1.

### Michaelis-Menten analysis

Since Michaelis-Menten parameters will depend on pH, ten individual Michaelis-Menten analyses were performed based on DMP oxidation, i.e. for both enzymes at pH 5, 6, 7, 8, and 8.5. The concentration range used for DMP was 0–12.5 mM. Data were analyzed in GraphPad Prism 6 using a non-competitive substrate inhibition model for the full substrate range or a regular Michaelis-Menten model for a truncated data set, giving overall similar results (see Figures S6 and S7 in File S1). Protein concentrations were estimated from the UV/VIS absorption maximum at 600 nm (specific to the T1 site). We used the extinction coefficient as previously reported for *B. subtilis* cotA of 4000 M^−1^cm^−1^ [40], which is similar to those of other bacterial laccases (e.g. 3900 M^−1^cm^−1^ [19]), making this assignment robust. Total protein concentration from the UV absorption at 280 nm (extinction coefficient 79300 M^−1^cm^−1^) suggested a purity of approximately 70%, consistent with minor impurities observed on the SDS-PAGE gel (Figure S2 in File S1).

A Michaelis-Menten experiment was performed at pH 8.5 (∼pH_opt_ for *B. clausii* cotA) using an apparent enzyme concentration of 1 nM. The resulting data were used to derive the kinetic data, based on an estimated protein concentration in the main kinetic analysis of 10 nM. When calculating this number, we accounted for the heat activation described later in this paper using a factor of 3.

### Investigation of salt effects

The effect of NaCl concentration on enzymatic catalysis was investigated in a series of measurements using variable salt concentrations and a pH range of 6–9. Final concentration of buffer was 50 mM, whereas NaCl was varied between 0–1 M. The enzyme was applied to a 96-well microtiter plate, and the enzyme assay was initiated by addition of SGZ to a concentration of 50 µM. SGZ was dissolved in methanol, and to reduce evaporation, fresh SGZ was used as retracted from a sealed volumetric flask for each assay. Activities were monitored as the initial rate of product formation, quantified from the absorption change at 530 nm.

## Results and Discussion

### The cotA gene from *B. clausii*

This study aimed to identify a protein that extends the positive characteristics of the *Bacillus subtilis* cotA in comparison to fungal laccases, with particular focus on activity and stability at alkaline pH. The impressive characteristics of *B. subtilis* cotA are surprising in the context of the native host being a mesophile, and we thus aimed at identifying orthologs from more extremophilic hosts. The cotA production is a late process of the *Bacillus* sporulation response, which is induced during stress conditions, but since sporulation is limited by the same factors as growth [41], the optimal growth conditions were used to identify high pH stability of related orthologs.

*B. clausii* KSM-16 encodes a cotA gene, has optimal growth at pH 9 at 40°C [38], compared to optimal growth at pH 8 for *B. subtilis*, suggesting a slightly higher alkaline tolerance. Another well studied sporulation enzyme from *Bacillus* is the subtisilin type protease with pH optima ranging from 8–13 [42], which is used in several industrial applications. The M protease from *B. clausii* belongs to the high-alkaline subtisilin group with maximum activity at pH 12.3. In contrast, the subtisilin proteases from e.g. *B.subtilis, B. licheniformis*, etc. have pH optima at pH 9–10. These two biological observations suggest higher spore stability at higher pH values and prompted our cloning, expression, and characterization of *B. clausii* cotA.

**Figure 1:**
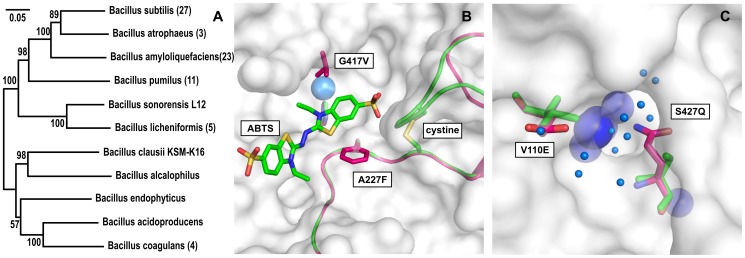
The cotA from *B. clausii*, compared to its ortholog from *B. subtilis*. (A) Nearest-neighbor tree of B. clausii cotA as described in the text. The bracketed number is the number of sequences represented by each node. Bootstrap values are given at the base of each branch. (B) The substrate binding pocket of cotA highlighting the differences between *B. subtilis* (green) and *B. clausii* (purple) cotA as described in the text. Amino acid changes are numbered on the *B. subtilis* cotA sequence and annotated with the *B. clausii* cotA amino acid last. (C) The water exit channel of cotA with similar highlighting. The T2 copper site is visible through the open channel. Small blue spheres represent water from pdb structure 3ZDW. Structures were visualized using PyMol 1.5 and the tree was generated using MEGA 5.

Figure 1A shows a sequence-derived nearest neighbor tree of homologous cotA enzymes created using MEGA 5.2 [43] started from the first 100 BLAST hits of *B. clausii* cotA against the Uniref90 database, and then reduced to only the high-scoring branches after a 1000-step bootstrap validation. It can be seen that the *B. clausii* cotA clusters together with a *B. alcalophilus* cotA in a different clade from that of *B. subtilis* cotA, showing a closer homology to this protein from an extremophilic bacteria. The *B. clausii* KSM-K16 cotA protein sequence was found to have 59% identity and 74% homology (BLOSUM62) with *B. subtilis* cotA, with most differences positioned within surface loops. Also, the network of ion pairs are not generally conserved between the two sequences (see Figure S1 in File S1). The most notably difference between the two primary sequences is that *B. clausii* cotA lacks the cystine forming loop present in *B. subtilis* cotA. Previously characterized cotA orthologs have all contained this cysteine bridge of unknown function [44].

Figures 1B and 1C show the superpositions of a structure of *B. clausii* from homology modeling (purple) on the *B. subtilis* cotA structure 3ZDW [45], (green). The tertiary structure of *B. clausii* cotA was constructed with Phyre2 [46], giving a homology model that was overall similar in tertiary and secondary structure to structure 3ZDW of *B. subtilis* cotA. Figure 1B shows the substrate binding pocket of cotA in complex with ABTS (3ZDW). The two loops that are connected by the cystine bridge in *B. subtilis* cotA are generally similar for the two proteins, apart from the tight GCGGD turn that allows the cysteines to bind. Two residues in contact with the substrate are substituted in *B. clausii* cotA; G417V and A227F, using the residue numbering from *B. subtilis* cotA. Both of these changes reflect substitutions from small aliphatic to bulky hydrophobic side chains, and they are positioned on opposing sides of the substrate binding pocket and close to the catalytic site.

Figure 1C shows the superposed water exit channels. Laccases use oxygen as co-substrate and produce water, both of which need to be transported via channels to the interior T2/or T3 copper sites, and thus variations in these part of the proteins could affect turnover. Two variations occur in sites that flank this channel, i.e. S427Q and V110E. There are no significant differences within the oxygen entrance channels of the two protein structures.

### Activity studies and pH optima

**Figure 2:**
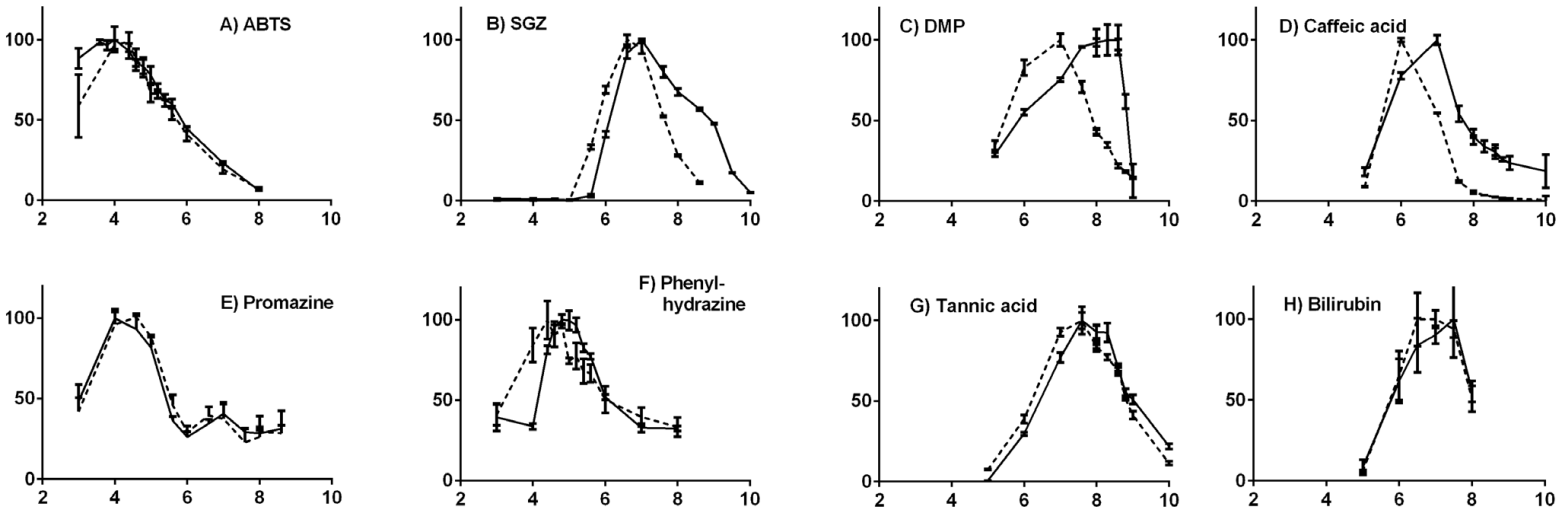
Activity vs. pH of the cotA from *B. clausii* (solid lines) on eight substrates, compared with that of cotA from *B. subtilis* (broken lines). Activities are averages with standard deviations from triplicate measurements of initial rates of product formation, corrected for buffer effects (see text) and normalized to 100. (A) ABTS; (B) SGZ; (C) DMP; (D) Caffeic acid; (E) Promazine; (F) Phenylhydrazine; (G) Tannic acid; (H) Bilirubin.

The pH-dependent activities were measured as initial linear rates, averaged over triplicate measurements for both laccases, using eight different substrates in the pH-range of 3–10: ABTS, SGZ, DMP, caffeic acid, promazine, phenyl hydrazine, and the more bulky tannic acid and bilirubin: Apart from hydrazine, all these substrates have previously been shown to be oxidized by some cotAs [16], [18]. The data are shown with standard deviations in Figure 2. Since we used three standard buffers to cover the studied pH intervals, we could identify a direct effect of buffer on the activity that has generally been ignored in previous work: Proteins prepared in the high-pH buffer CHES displayed higher activity for the same pH (8.6) than the medium-range buffer MOPS, and MOPS gave a higher activity than the low-pH range McIlvaine buffer at pH 8; the buffer systems affected rates up to ca. 40% of total activity. We thus performed multiple measurements at the limiting, common pH values of the buffers (full data in Figure S3 in File S1); the data presented in Figure 2 are corrected for the buffer effect by scaling the data points of the high-activity buffers to align with data points from the McIlvaine buffer at their common pH values.

We first compare our obtained activity of the cotA from *B. subtilis* with previous work, in particular the pH optima with ABTS, SGZ, and DMP [15], [35]. As seen in Figure 2A, the ABTS oxidation rate of cotA from *B. subtilis* has maximum near pH∼4. This value is consistent with the pH_opt_(ABTS) of ∼4 obtained previously for cotA from *B. subtilis* [35]. Furthermore, the activity behavior vs. pH is quite similar for the new cotA across the pH range, given similar pH_opt_ for this substrate.

The relative activity on SGZ is shown in Figure 2B. While other phenolic substrates exhibited substantial auto-oxidation, to be discussed below, we found no measurable auto-oxidation of SGZ in the pH-range 3–10 despite its phenolic ring system. The difference could indicate that the abstracted SGZ electron is not localized on the hydroxy group but more likely the azo group. The pH_opt_∼7 is also consistent with that reported previously for *B. subtilis* cotA [35]. The notable difference between the two enzymes is that the laccase from *B. clausii* remains active at a higher pH than its ortholog from *B. subtilis* and keeps 50% of the activity at pH 9 at which the *B. subtilis* cotA displays almost no activity.

In terms of the phenolic substrate DMP (Figure 2C), we found an optimal turnover for *B. subtilis* cotA at pH∼7, which is in agreement with previously reported pH_opt_ for this protein and substrate [35]. In contrast, the cotA from *B. clausii* was most effective above pH 8, showing a significant shift in pH_opt_ (∼1.3) compared to the cotA from *B. subtilis*, further indicating enhanced alkalophilicity of the cotA from *B. clausii*.

Caffeic acid is a dihydroxy cinammic acid and is one of the major natural oxidants mainly found in leaves. It is a general phenolic laccase substrate [16], but without the substituted methoxy groups of SGZ and DMP. The first phenolic proton has a pK_a_ 8.6, but the compound is already charged through the full pH range from deprotonization of a carboxylic acid. The pH activity profile (Figure 2D) shows an optimal pH shifting from 6 for *B. subtilis* cotA to 7 for *B. clausii* cotA. Similar to SGZ, the activity of *B. clausii* cotA broadens into the alkaline range with 40% activity at pH 8 compared to none for *B. subtilis* cotA.

We also investigated the activity of the two enzymes on promazine, a synthetic non-phenolic phenothiazine drug. The observed oxidation was not catalyzed by 1 mM CuCl_2_. In a large screening of laccase substrates, promazine was previously found to be oxidized by laccases of bacterial but not fungal origin [16], making it an interesting substrate to probe potential new oxidation capabilities of bacterial laccases. As seen in Figure 2E, the two enzymes displayed very similar activities across pH for this substrate against a high background of auto-oxidation (full data in Figure S3 in File S1). The pH_opt_ was found to be in the range 4–5 for both enzymes on this substrate, i.e. slightly higher than for ABTS.

We further tested activity of the enzymes on phenylhydrazine, a potent reducing agent with E°  = 410 mV [47] that readily reacts with Cu(II) (Figure 2F). It has previously been described as both an inhibitor and substrate for a tyrosinase [48], ultimately resulting in an *o*-quinone. Our reaction with laccases produced a similar UV-VIS spectrum, indicating that the same reaction occurs, and both laccases were found to oxidize this compound. The laccase from *B. clausii* and *B. subtilis* were found to have slightly different pH_opt_ shifting from ∼4.5 in *B. subtilis* cotA to ∼5 with *B. clausii* cotA.

We also investigated the activity on an unusual phenolic substrate, tannic acid (Figure 2G). Tannic acid is a plant tannin consisting of branching polygalloyl sugar esters. The tri-hydroxo phenyl moieties make the compound acidic with a first pK_a_ of ∼4 [49], and it is a potent metal chelator. In addition, tannic acids precipitate proteins and are considered a general plant defense against herbivores and bacteria. Tannic acids have been reported to inhibit tyrosinases [50]. The optimal pH activity profiles for oxidation of tannic acid (Figure 2G) are similar for both enzymes, with pH optima of ∼7.5±0.5, although the curve of *B. clausii* cotA shifted ∼0.5 pH units towards more alkaline conditions.

Finally, to further expand the chemical diversity, we also investigated the activity on bilirubin, which has been shown to be a substrate for several laccase-like enzymes, and also for cotA [18]. The results (Figure 2H) indicated a similar upper and lower pH for activity for the two enzymes, and the exact pH_opt_ are similar within the uncertainties of the assay and comparable to that previously reported [18]. Thus, the bilirubin assay confirms that the alkaline advantage of the new cotA mainly manifests in the phenolic substrates.

An important factor affecting activity across pH is the changing difference in redox potential between the substrate and enzyme [33]. For each decreased pH unit, the difference in redox potential will increase by 60 mV viz. the Nernst equation, driving oxidation up [33]. Another effect comes from the pK_a_ value of phenolic substrates whose deprotonized forms have much lower redox potentials, also accelerating oxidation when pH increases. These two activity-increasing effects at low and high pH are countered at extreme pH by protein stability and at high pH, the direct OH^−^ inhibition, most likely due to OH^−^ binding to the T2/T3 site. However, these effects, general to all laccases, cannot explain the protein-specific differences that we observe. The decreasing redox potential of proton-labile substrates with increasing pH also gives rise to a substantial auto-oxidation at higher pH values (see Figure S3 in File S1).

There are three important trends in the pH profiles. First, promazine, ABTS, bilirubin, and to some extent phenylhydrazine and tannic acid show similar activity profiles vs. pH for the enzymes, with similar pH_opt_, despite some enhanced loss of *B. clausii* cotA activity at low pH for ABTS and phenylhydrazine. These substrates are not simple phenols and perhaps except phenylhydrazine, they do not change charge in the measured pH range.

Second and in contrast, caffeic acid, SGZ, and DMP (and to some extent the polyphenol tannic acid) all have their active pH range increased for *B. clausii* cotA, compared to *B. subtilis* cotA. The pK_a_ value of these compounds are 8.6, 8.8, and 10.0 [51], [52], i.e. they change charge in the monitored pH range. Interestingly, the pH optima follow the pK_a_ values with 7, 7, and 8 for *B. clausii* cotA and 6, 6.6, and 7 for *B. subtilis* cotA, with a general trend of the *B. clausii* laccase being more active at higher pH.

A third trend, potentially related to the second one, is a shoulder in the activity profile in the pH range 8–9 for *B. clausii* cotA, which makes the activity profiles skewed towards higher pH. This is seen for SGZ, caffeic acid, DMP and to some extent for tannic acid, showing that this is a protein-specific effect. As this effect was seen for all the measured substrates that have activities in this pH range and since the effect did not follow the pK_a_ of substrates, it suggests that *B. clausii* cotA has adapted to a generally more alkaline environment, consistent with an optimal growth at pH 9 of its host organism [38]. This non-substrate-specific activity effect coincides with the emerging OH^−^ inhibition at above-neutral pH. Figure 1C shows the structural changes around the water exit channel of the two cotAs. *B. clausii* cotA has a glutamic acid (V110E) on the brim of the exit channel, which in itself, via electrostatic repulsion, might reduce OH^−^ affinity for the channel, or it can possibly make an interaction with the other variable amino acid 427Q to reduce OH^−^ entry during catalytic turnover. This mechanism would be similar to that suggested for an alkali-tolerant mutant of *T. versicolor* laccase derived from directed evolution, which had a N109S mutation suggested to affect OH^−^ entry [7].

In conclusion, the activity of the newly characterized cotA from *B. clausii* is in some ways similar to that of the cotA from *B. subtilis*, but its increased pH_opt_ and the broadened activity profiles up to 1–2 units higher pH for the phenolic substrates DMP, caffeic acid, and SGZ suggest a mechanism of alkali-tolerance that is explored further below.

### Incubation at variable pH and T: Activation processes and stability

The stability of bacterial laccases is one of the key properties rendering them attractive in diverse applications where protein life time is of major importance. To investigate the stability of the newly characterized cotA from *B. clausii* we first measured the activity of protein samples that had been stored in buffers at pH 3–10 and incubated at 30°C and 50°C for five hours (See Figure S4 and Figure S5 in File S1). From these experiments, the stability of *B. subtilis* cotA appears to be maximal in the range 7.6–8.6, while the *B. clausii* cotA shows a monotonic increase in activity as the pH becomes more alkaline. Data suggests that there is a significant difference in the interplay between pH and thermostability between the two enzymes. A similar profile of residual activity after incubation has also recently been seen for *Streptomyces sviceus* laccase [10].

**Figure 3:**
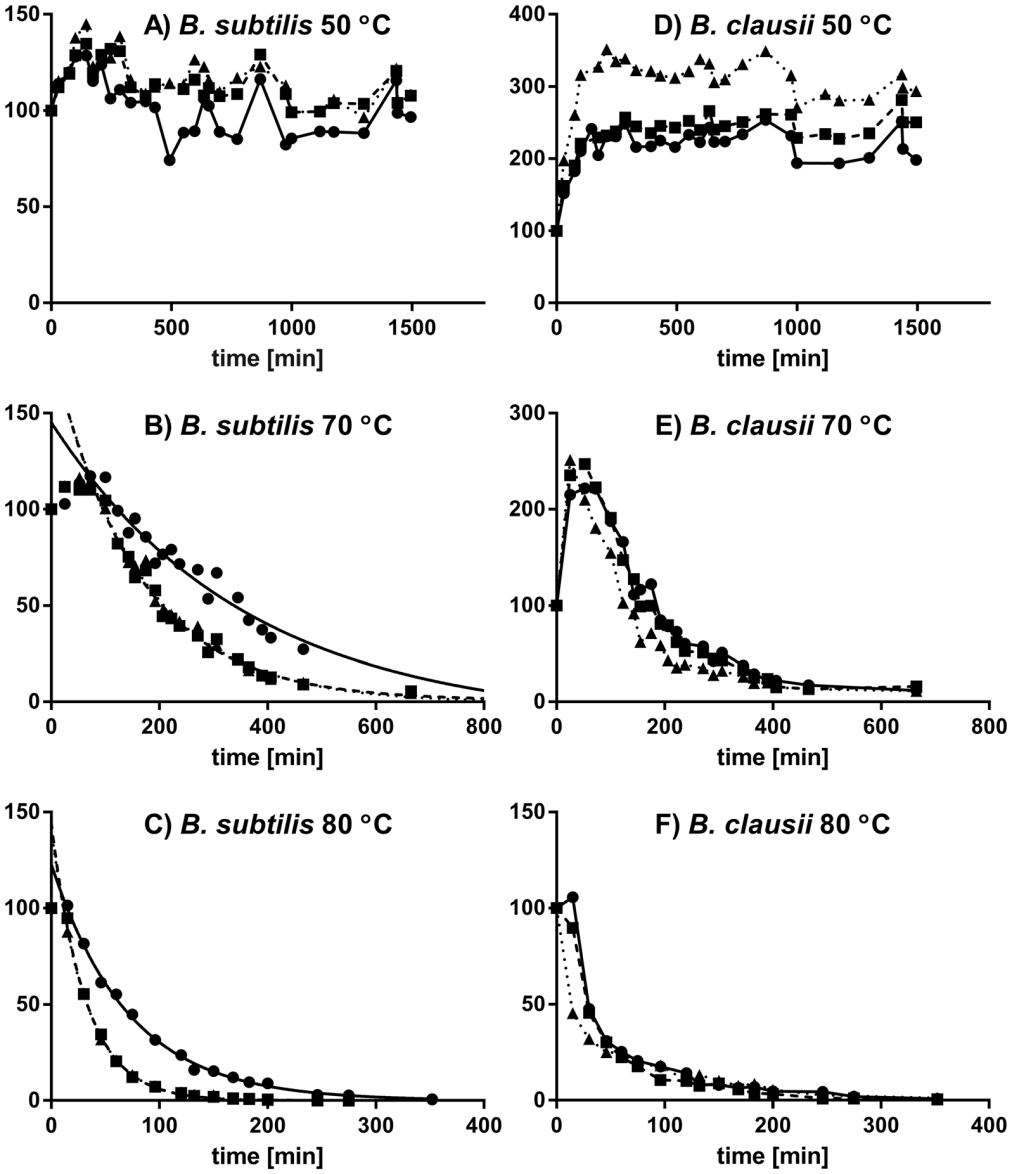
Stabilities of the cotAs from *B. clausii* and *B. subtilis* vs. temperature and pH 8 (circles, solid line), 9 (squares, broken line) and 10 (triangles, dotted line). (A) *B. subtilis* cotA at 50°C; (B) *B. subtilis* cotA at 70°C; (C) *B. subtilis* cotA at 80°C; (D) *B. clausii* cotA at 50°C; (E) *B. clausii* cotA at 70°C; (F) *B. clausii* cotA at 80°C. Activities were normalized to the starting activity before heat incubation.

**Figure 4:**
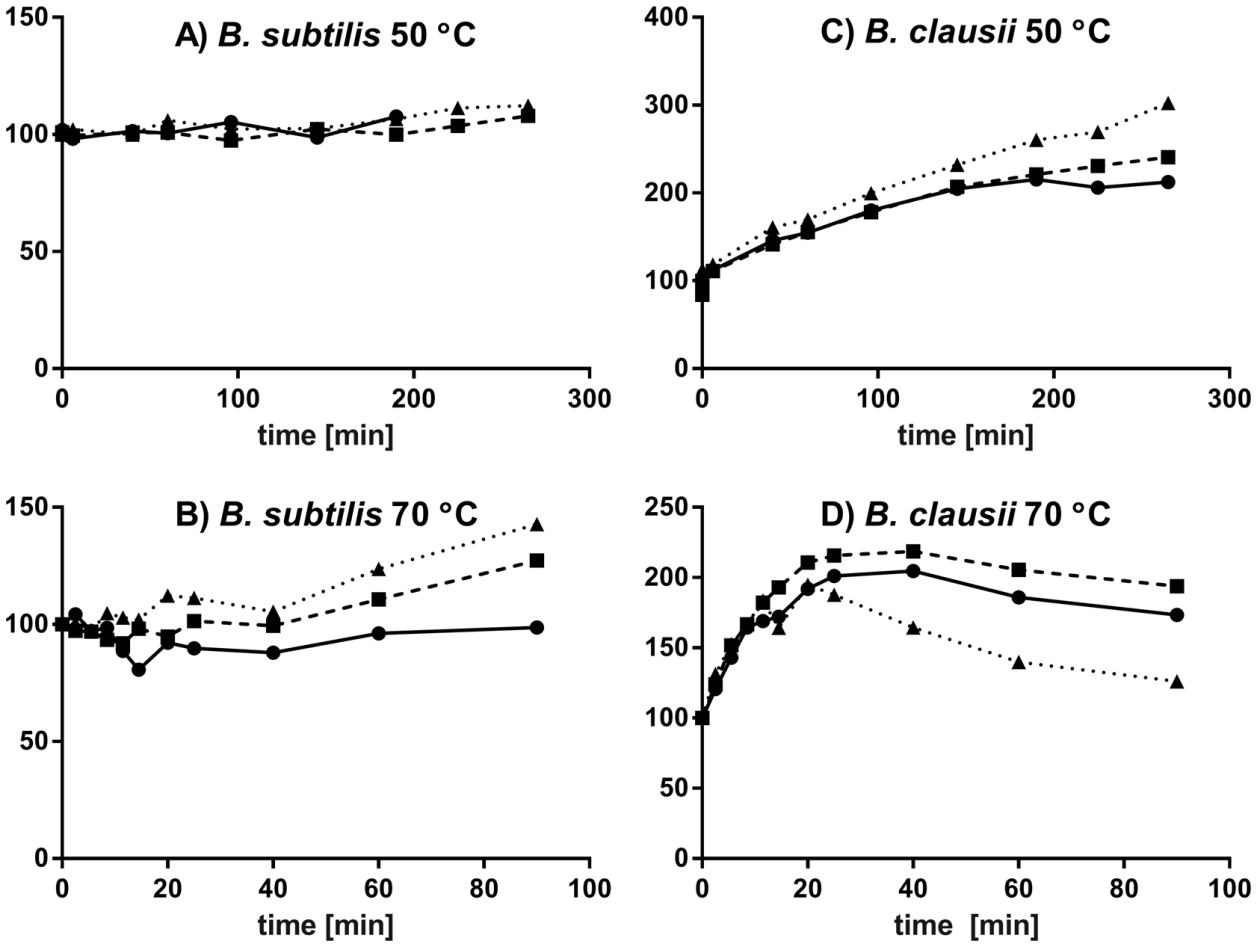
Short-term residual activity of the proteins after incubation. Heat activation is visible at pH(circles, solid line), 9 (squares, broken line) and 10 (triangles, dotted line). (A) *B. subtilis* cotA at 50°C; (B) *B. subtilis* cotA at 70°C; (C) *B. clausii* cotA at 50°C; (D) *B. clausii* cotA at 70°C.

To investigate the temperature behavior of the newly characterized cotA from *B. clausii* in more detail, we studied its residual activity after incubation at various times at 50°C, 70°C, and 80°C, as described in the Methods section. Again, we performed all measurements completely in parallel also for the cotA from *B. subtilis*, to enable a strict benchmark for characterization. Furthermore, we did all the incubations and the subsequent activity measurements at three pH values, 8, 9, and 10, because pH variations, although rarely reported, markedly influence residual activity or apparent thermostability upon incubation, and we had observed that pH significantly affects the relative apparent stability of the two enzymes. The combined data for these measurements are shown in Figure 3 and Figure 4. We found thermal activation at short incubation times (shorter for higher temperature) before thermal protein degradation dominated the subsequently measured initial rates. This was especially prominent for *B. clausii* laccase and complicates the analysis of the thermostability.

Figure 3 shows the longer-time-frame data of thermal incubation at variable pH. At 50°C (Figure 3A, 3D), none of the proteins displayed any significant degradation within the first 1500 minutes of incubation. At 70°C (Figure 3B, 3E), the degradation of protein was clearly evident, but so was heat activation and the fate of the proteins is a non-trivial mixture of protein degradation and heat activation. An attempt to fit an exponential decay to the *B. subtilis* cotA data is given in Figure 3B, where only the data after 120 minutes have been used in the fitting procedure. *B. subtilis* cotA show best stability at pH 8 with an approximate t_½_(70°C) of 250 minutes compared to 110 minutes at pH 9 and pH 10. A similar fit was not possible for *B. clausii* due to its substantial heat activation but it is clear that its stability at pH 8 and 9 is slightly higher than at pH 10. At 80°C (Figure 3C, 3F), there is no visible thermal activation, because the degradation is faster than the heat activation. Still, a plateau resulting from combined activation and degradation is visible within the first 20 minutes for both laccases except at pH 10 for cotA from *B. clausii*, which is degraded quickly at this high temperature. The exponential decay function for *B. subtilis* cotA where the first 20 minutes have been disregarded in the fitting procedure is given in Figure 3C. The t_½_(80°C) was measured to be 50 minutes at pH 8 and 22 minutes at pH 9 and pH 10. This compares well with the 56 minutes that can be read from the data in the original *B. subtilis* cotA characterization from 80°C incubation at pH 7.6 [15]. We estimated the corresponding t_½_(80°C) of *B. clausii* cotA to be ∼20 minutes at pH 8 and substantially less pH-dependent than cotA from *B. subtilis*.

From all measurements, *B. clausii* cotA had similar apparent stabilities in pH 8 and pH 9 and slightly lower stability in pH 10. In contrast, *B. subtilis* cotA exhibited highest apparent stabilities at pH 8. The stabilities are best compared at 80°C where heat activation does not substantially affect the short-time data. At pH 8 *B. subtilis* cotA has 30% activity after 100 minutes of incubation at 80°C. A similar reading of *B. clausii* cotA at pH 9 gives 18%. These data thus show that the new cotA from *B. clausii* is slightly less thermostable than the cotA from *B. subtilis*, but has traded some of this thermostability for a higher stability at pH 9–10, whereas cotA from *B. subtilis* is found to be clearly most stable at pH 8.

From the short-time interval measurements shown in Figure 4, we can see that at 50°C, cotA from *B. subtilis* (Figure 4A) displayed no thermal activation at any pH whereas the laccase from *B. clausii* (Figure 4C) was activated three-fold within the first 300 minutes at pH 10, and two-fold at pH 8. The unusual, higher activation at higher pH for *B. clausii* cotA was consistent across the measured rates at pH 8, 9, and 10, and thus significant. At an incubation temperature of 70°C, the cotA from *B. subtilis* began to display some thermal activation up to 140% at pH 10, but no activation at pH 8 (Figure 4B). At 70°C, the cotA from *B. clausii* (Figure 4D) displayed activation up to 220% within 20–40 minutes and then started to degrade. These data show the importance of pH for reporting thermal activation and thermostability.

We found a general trend for both cotAs of more heat activation at higher pH. This is not a result of higher stability which levels off below pH 10 as discussed previously, and must thus be a true property of the intrinsic cotA activation. Thermal activation is commonly observed for laccases and is not generally understood [53]. However, it is likely to either imply a removal of partly inhibiting molecules from substrate binding sites or channels prior to the first catalytic cycles, or alternatively, involve a protein conformational change, where the active form of the protein is (entropically) favored at higher temperature. Alternatively, we suggest that heat favors release of small inhibitors or water in the channels of the enzymes, as their release would be entropically favored and thus more pronounced at high temperature. This heat-favored release may further be more pronounced in proteins with specific residues in the channels that favor anion release, such as the negative charge of *B. clausii* cotA.

### Activation and inhibition of cotA vs. pH and NaCl concentration

Laccases are well-known to be inhibited by small anions, as observed for fungal laccases at low pH [2], [3]. However, there are also previous reports that NaCl can activate proteins, as observed in another alkalophilic bacterial laccase [22], which displayed pH_opt_ values of 7.9 for SGZ and 7.5 for DMP [54]. This effect seems to be complex and pH-dependent, as discussed previously: OH^−^ and Cl^−^ are likely to compete as inhibitors at the T2/T3 site of a laccase, as has been observed for fluoride [33], [55]. Also, both the Na^+^ and Cl^−^ ions may affect the stability and turnover in some pH ranges due to non-specific electrostatic interactions with the proteins, e.g. via Hoffmeister effects. Thus, to optimize catalytic performance, it is of importance to understand how NaCl and pH play together to activate or inhibit laccase activity.

**Figure 5:**
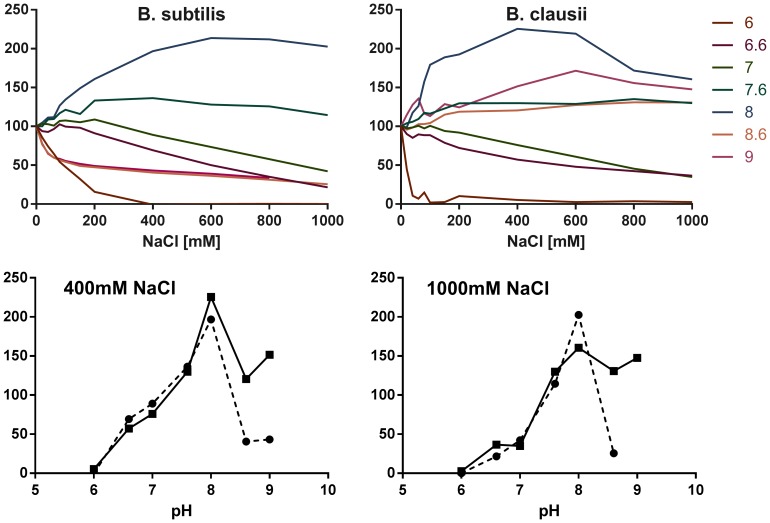
Activity of cotA from *B. subtilis* and *B. clausii* as a function of NaCl concentration and pH. (A) *B. subtilis* cotA; (B) *B. clausii* cotA; (C) Intersection at 400 mM NaCl and (D) intersection at 1000 mM NaCl, with *B. clausii* cotA in full lines and *B. subtilis* cotA in broken lines.

Figure 5 shows the activity (initial rates of SGZ product formation) of cotA from *B. subtilis* and *B. clausii* as a function of *both* NaCl concentration and pH. It can be seen from Figures 5A and 5B that both proteins are inhibited at acidic pH, as observed before for F^−^ [33], [56] and typically seen in fungal laccase assays where activity is highest at low pH [53]. However, we also observe a substantial *activation* of both proteins at higher pH, even though these measurements were done at room temperature.

To further confirm the estimates of the alkali-tolerance of the proteins, the intersections at 400 mM NaCl and 1000 mM NaCl are shown in Figures 5C and 5D, respectively. From these figures, it is more clear that the cotA from *B. subtilis* loses activity quickly above pH 8, whereas the cotA from *B. clausii* preserves substantial activity at higher pH, consistent with the results from the activity profiles vs. pH, Figure 2.

The most important conclusion from these experiments seems to be that chloride inhibition of laccases at low pH is countered at high pH by activation, meaning that higher pH is very desirable also for this reason in laccase applications. It is reasonable that chloride inhibition would be electrostatically favored at low pH where the protein channels are likely less negatively charged, if electrostatics affects the OH^−^ and Cl^−^ affinity. The strong dependence of halide inhibition on pH means that the halide tolerances of different laccases, which are commonly described at only one pH and compared to each other at variable pH [57], should be quantified over a range of pH to be commensurable.

Heat activation is common for laccases, but for *B. clausii* we also observed similar effect from high pH incubation and NaCl. These three observations can potentially be derived from the same mechanism. A simple explanation is that the laccases are produced in a non-functional form in the heterologous host. However, activation has been observed up to 150% for natively expressed cotA in *B. subtilis* spores at 80°C [15], and activation up to 130% has been observed at 37°C for *B. pumilius* laccase [58], which is within the optimal temperature range for growth of the native host. Thus, it seems likely that cotA activation occurs *in vivo* as part of the function of this enzyme in the sporulation process.

Introduction of a lysine into *B. subtilis* HR03 CotA [59] has been shown to significantly increase the potential heat activation to an extreme 900%. This was explained by the introduction of a new ionic interaction between two domains. It was hypothesized that this interaction is broken at high temperature, creating a more active enzyme. A similar change in ionic interactions as pH approaches the pK_a_ of an important salt bridge might explain our observed enzyme activation at higher pH. Since NaCl activation occurred in combination with pH activation, it is possible that high ionic strength may destabilize labile salt bridges to give a similar response, but understanding the complex interplay of temperature, pH and salt on activation-inhibition of laccases clearly requires more mechanistic investigations.

### Variation in K_M_ and k_cat_ as a function of pH

**Figure 6:**
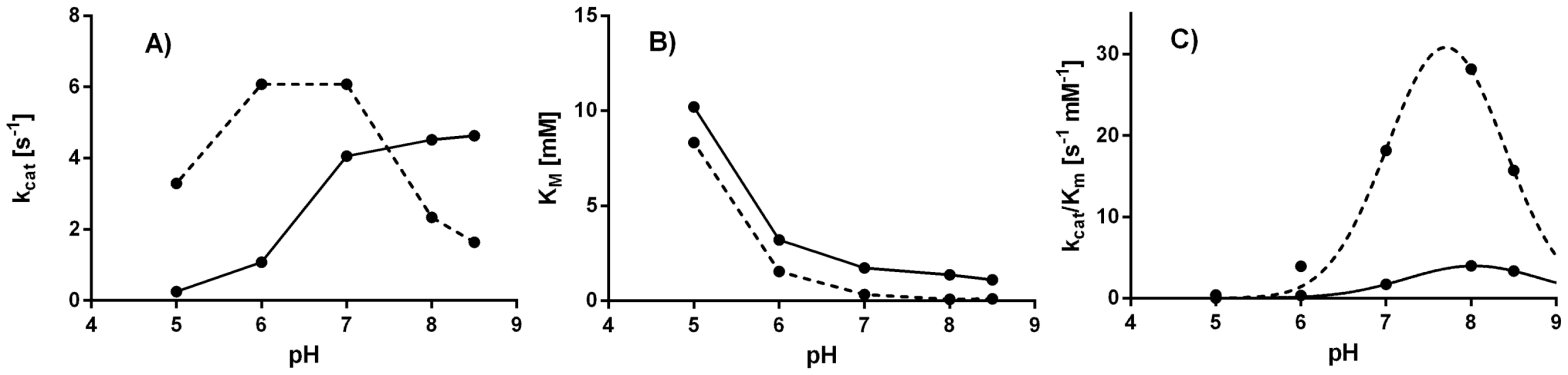
k_cat_ and K_M_ for DMP oxidation as a function of pH for *B. subtilis* cotA (broken lines) and *B. clausii* cotA (full lines). (A) k_cat_ in s^−1^. (B) K_M_ in mM (C) k_cat_/K_M_ showing the effect on total pH-dependent activity. The profiles were fitted with a Gaussian using a mean pH of 7.7 and an amplitude of 30.8 for *B. subtilis* cotA and 8.1 and 4.0 for *B. clausii* cotA, respectively. Please see File S1 for full data.

To understand the causes of the pH-dependent activity differences between the two enzymes, which is most pronounced in DMP (Figure 2), we additionally performed a Michaelis Menten analysis for both enzymes at pH values of 5, 6, 7, 8, and 8.5 (full data in Figures S6, S7, and S8 in File S1). Figure 6 shows the behavior of k_cat_, K_M_, and k_cat_/K_M_ as a function of pH resulting from this analysis.

All parameters vary substantially with pH, suggesting that the use of one pH for Michaelis-Menten analysis is insufficient. While the K_M_s behave largely similar vs. pH (Figure 6B), there are substantial differences in the pH-dependence of k_cat_ for the two enzymes (Figure 6A). The ratios of the two parameters depend similarly on pH (Figure 6C), although the optimum for cotA from *B. clausii* is at ∼0.4 higher pH, as obtained from a Gaussian fitting. From this, we conclude that the pH-dependent differences measured from the assays of phenolic substrates, including DMP, are largely due to differences in k_cat_, i.e. the electron transfer catalytic step, and not substrate binding (K_M_). This finding confirms that the performance of cotA from *B. clausii* at higher pH is enhanced by activity associated with the copper sites, not the substrate binding step, and is consistent with an enhanced ability to prevent anion inhibition. Our K_M_ derived from non-linear fitting at pH 7 was 0.34 mM for cotA from *B. subtilis*, somewhat larger than the 0.23 mM determined by Durao et al. [40] As outlined in the supporting information (Figures S6 and S7 in File S1), DMP kinetics give rise to substrate inhibition. Thus, the difference could result from substrate concentration effects or different amounts of active enzyme, which is a major challenge to laccase benchmarking. The measured K_M_ for *B. clausii* cotA was 1.02 mM, similar to cotA from *B. pumilus* with reported K_M_ 0.822 mM towards DMP at pH 7 and 37°C [58].

We observed substantial conversion between active and inactive forms of the enzymes, as evident also partly from the heat activation in Figure 3, rendering a definite assignment of active enzyme amount error-prone. We accounted for this activation in the calculation of protein concentration and k_cat_ for *B. clausii* using a factor of 3 as indicated by Figure 3 and used absorption at 600 nm for concentration estimation (File S1, Figure S8). The values of k_cat_ deduced from the analysis (Figure 6) are for *B. subtilis* cotA ∼6 s^−1^ at pH 6 and ∼2 s^−1^ at pH 8 and for *B. clausii* cotA 1 s^−1^ at pH 6 and 5 s^−1^ at pH 8. The reported k_cat_ of *B. subtilis* cotA is in the lower end of the previously reported range 7–30 s^−1^ at pH 7 [40]. The k_cat_/K_M_ curves generally follow the pH-dependent activities of Figure 2. The k_cat_/K_M_ values resulting from Gaussian fitting (Figure 6) are 10-fold higher for *B. subtilis* cotA, suggesting that the alkali-salt tolerance discussed above has been achieved at the expense of catalytic proficiency. A more detailed kinetic analysis requires a protocol to fully convert between inactive and active protein forms – We are currently exploring work in this direction.

In addition to conversions between active and inactive forms, auto-oxidation, and buffer effects, which should be carefully accounted for during laccase characterization, we also observed a substantial non-competitive inhibition of both enzymes starting at DMP concentrations of ∼2.5 mM. The Michaelis-Menten data were thus derived also from non-linear fitting taking inhibition into account, given similar results (See Figure S6 and Figure S7 in File S1, last panels). Moreover, since we performed the Michaelis-Menten analysis at various pH-values, we also observed that this inhibition is absent at acidic pH but initiates at pH∼6 for *B. subtilis* cotA and at pH∼7 for *B. clausii* cotA. This inhibition further increases with increasing pH. Thus, the pH-dependence of the non-competitive inhibition is also consistent with the alkaline advantage of cotA from *B. clausii*.

## Conclusions

We have reported the cloning, expression and characterization of a cotA from *B. clausii*, strictly benchmarked against its well-known ortholog from *B. subtilis*, expressed heterologously and characterized fully in parallel. We find that cotA from *B. clausii* has a shift in optimal pH for oxidation of phenolic substrates and a high salt resistance at high pH, compared to its ortholog from *B. subtilis*. We also identify important, pH-dependent effects of buffers, auto-oxidation, thermal activation, and non-competitive substrate inhibition that, together with the general changes in relative redox potentials of substrates and laccases, confirm and expand the previously noted [60] complex behavior of these enzymes under variable pH and NaCl concentration.

The stabilities of the two enzymes were substantial, with half-times of inactivation of 50 and 20 minutes for cotAs from *B. subtilis* and *B. clausii*, respectively, at pH 8 and 80°C. In comparison, the newly reported alkali-stable laccases from *actinomycetes* [12] and *Trametes sp.* [11] had similar half-lifes (60 minutes) at only 60°C and pH 8, showing the substantial advantage of the characterized bacterial laccases over fungal orthologs if stability is preferred over redox potential. This advantage is, as shown in this work, further enhanced by the interplay of salt and pH at alkaline conditions where the cotA enzymes, in particular the cotA from *B. clausii*, work well.

The additional proficiency of cotA from *B. clausii* could be due to substitutions relative to its *B. subtilis* ortholog that may repel anion inhibitors in the water exit channel (notably V110E, Figure 1), although this requires further investigation, as does the complex interplay between pH and salt. From Michaelis Menten analysis, we found that the alkaline activity shift of the new cotA is not due to K_M_ but mainly to k_cat_, suggesting changes at the copper sites rather than the T1/substrate binding sites. While this shift is consistent with higher alkali-salt tolerance and potentially explained by increased anion repulsion in the water exit channel, this tolerance has been at the expense of 10-fold lower k_cat_/K_M_ (DMP) compared to cotA from *B. subtilis*. The observed trade-off in the new cotA between alkaline stability and catalytic proficiency should be of interest when optimizing bacterial laccases for applications under neutral or alkaline conditions.

## Supporting Information

File S1 **File S1 contains supporting data on sequence alignment of cotA orthologs (Figure S1), SDS page (Figure S2), activity profiles vs. pH without correcting for buffer differences and without subtraction of auto oxidation (Figure S3), data for pH-stability from activity measurements after incubation (Figure S4 and Figure S5), and data for the Michaelis Menten kinetics of cotAs from** ***B. subtilis*** **(Figure S6) and** ***B. clausii*** **(Figure S7). UV-VIS absorption profile of** ***B. clausii*** **cot A. (Figure S8).**
(PDF)
