# Supplementary material for: Characterization of an Alkali- and Halide-Resistant Laccase Expressed in E. coli: CotA from Bacillus clausii
Source: PLoS One. 2014 Jun 10;9(6):e99402. doi: 10.1371/journal.pone.0099402 (PMC4051777; doi:10.1371/journal.pone.0099402)

Supporting Information

Characterization of an alkali- and halide-resistant laccase expressed in *E. coli*: CotA from *Bacillus clausii*

Søren Brander[a,b], Jørn D. Mikkelsen[a], Kasper P. Kepp[b]*

[a] *Technical University of Denmark, DTU Chemical Engineering, DK 2800 Kongens Lyngby, Denmark*

[b] *Technical University of Denmark, DTU Chemistry, DK 2800 Kongens Lyngby, Denmark*

* Corresponding author: E-mail: kpj@kemi.dtu.dk. Phone: +45 45 25 24 09.

Figure S1. Sequence alignment of cotA from *B. clausii* vs. close orthologs.Sequence alignment was performed using the guidance server (MAFFT algorithm). Alignment has been visualized in clustalX.

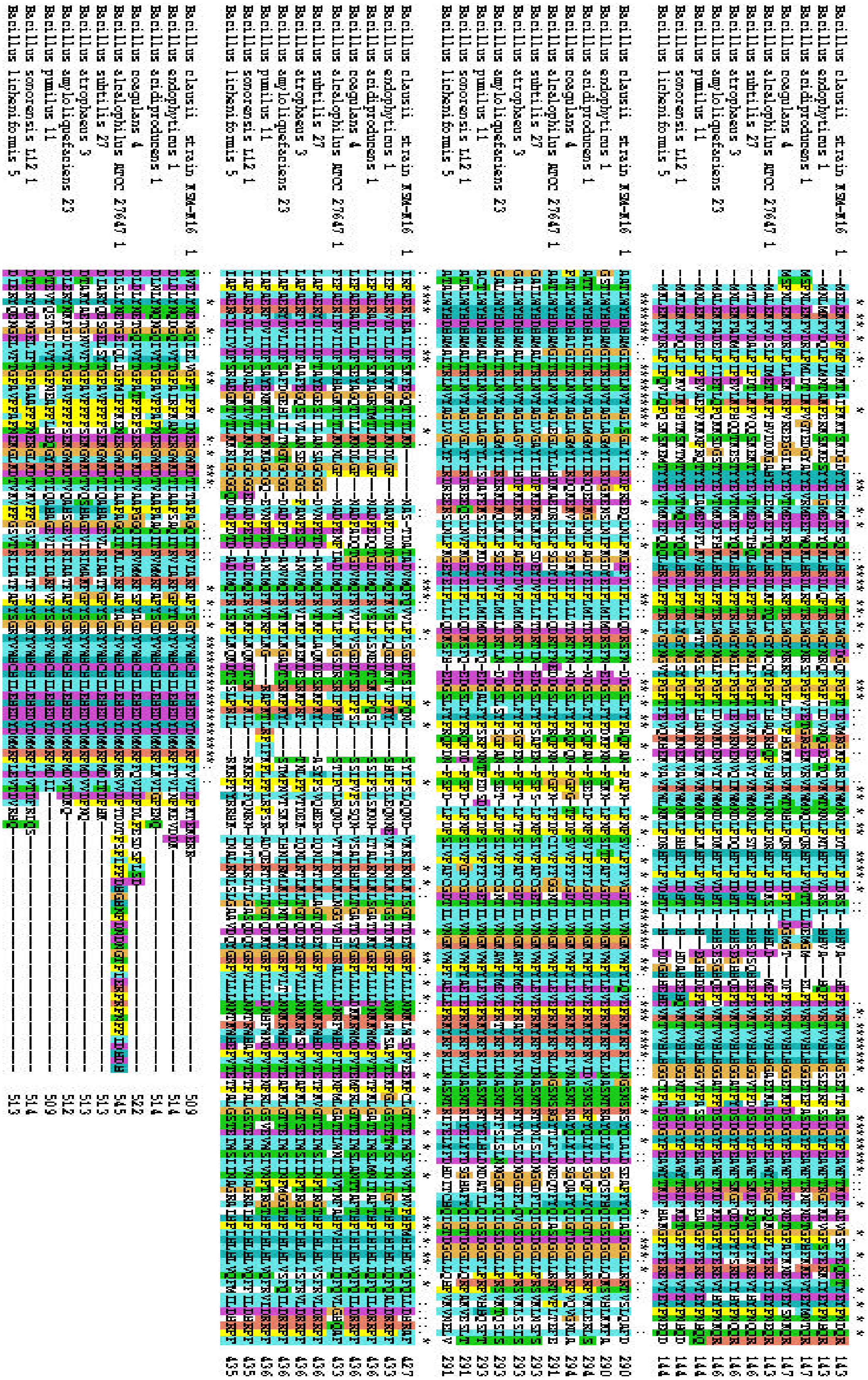

Figure S2. SDS PAGE gel of purified *B. clausii* cotA. Lane 1 is the purified and upconcentrated protein. Lane 2 is a 20-times dilution of Lane 1. Lane 3 is a Precision Plus Pre-stained protein standard (BioRAD), and the dotted line represents lanes that has been edited out.

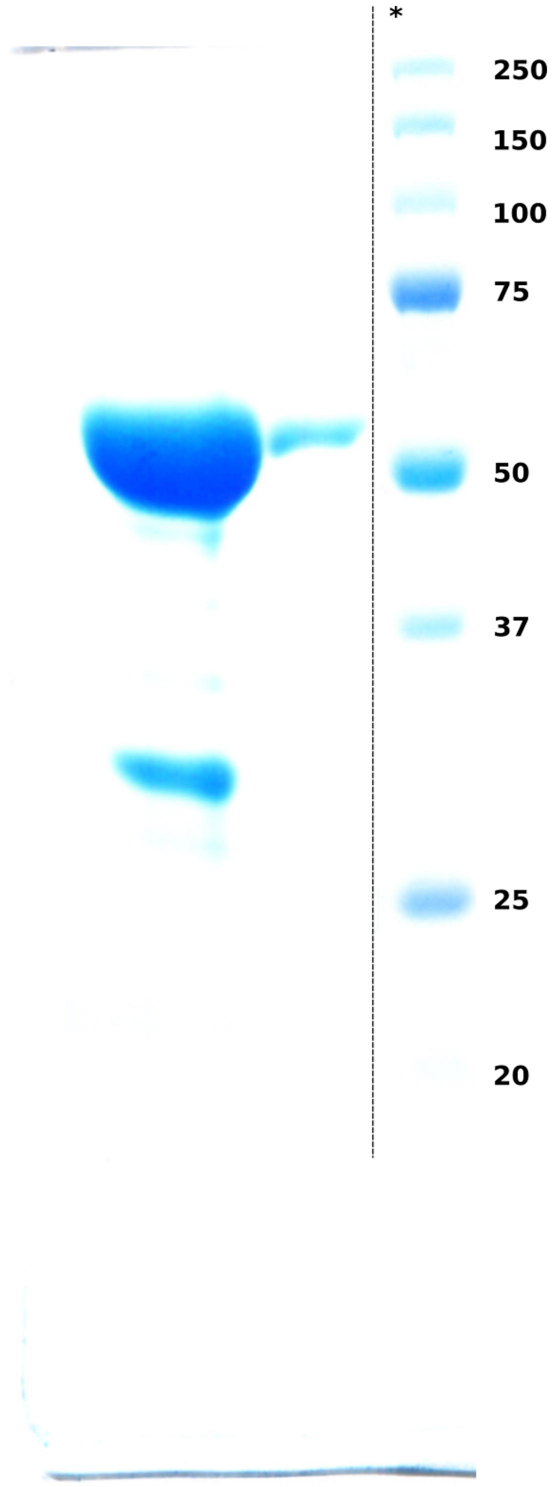

Figure S3. Activities vs. pH of *B. subtilis* (red), *B. clausii* (blue) cotAs on eight substrates, including auto-oxidation profiles (green). Data is presented without substraction of auto-oxidation, and without correction for buffer differences.

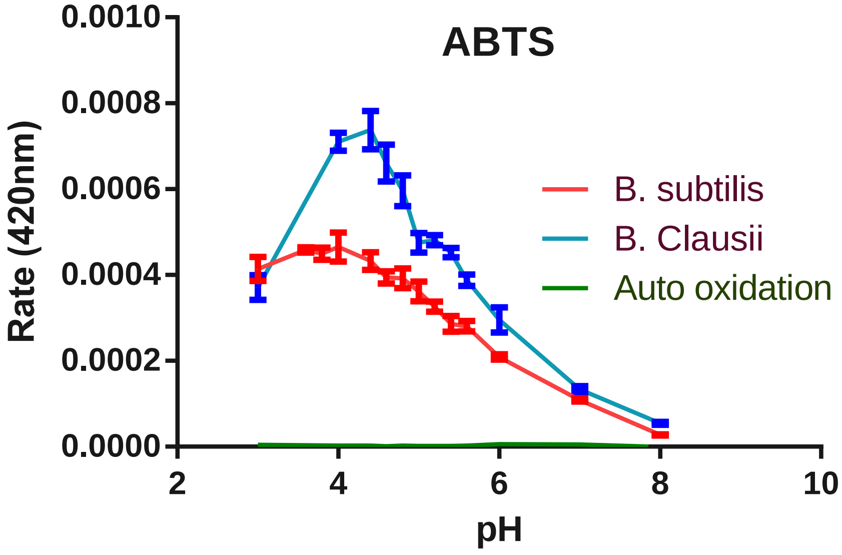

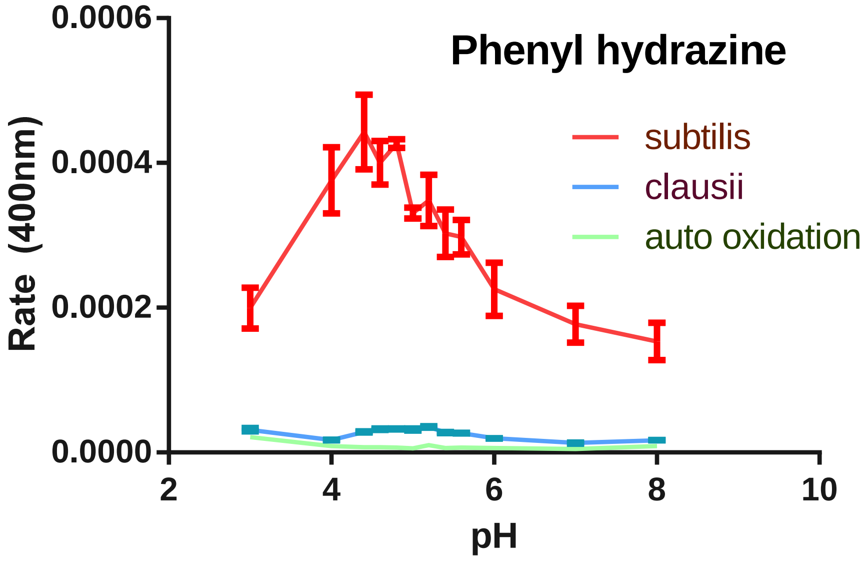

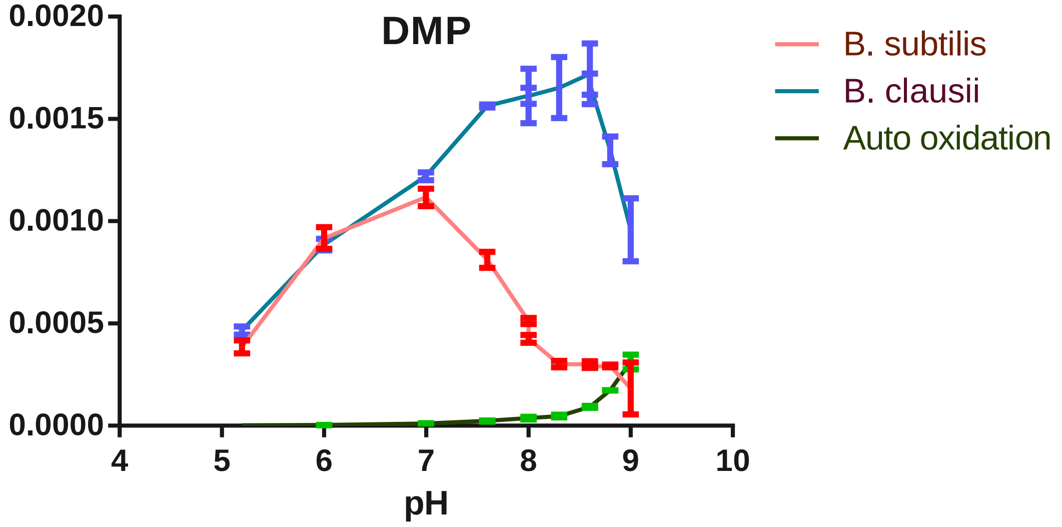
DMP
0.0020
0.0015
0.0010
0.0005
0.0000
4
5
6
7
8
9
10
pH
B. subtilis
B. clausii
Auto oxidation

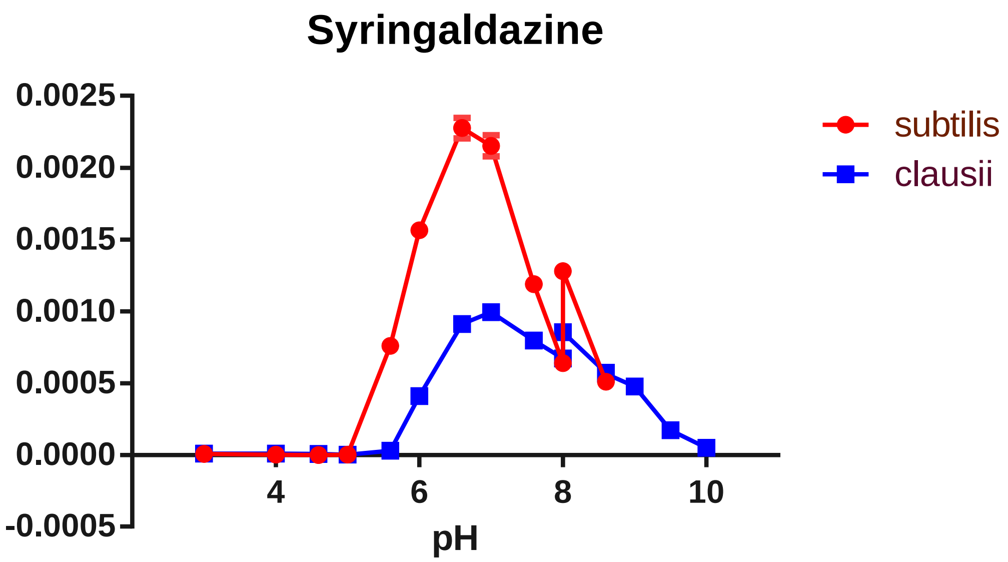
Syringaldazine
0.0025
0.0020
0.0015
0.0010
0.0005
0.0000
-0.0005
4
6
8
10
pH
subtilis
clausii

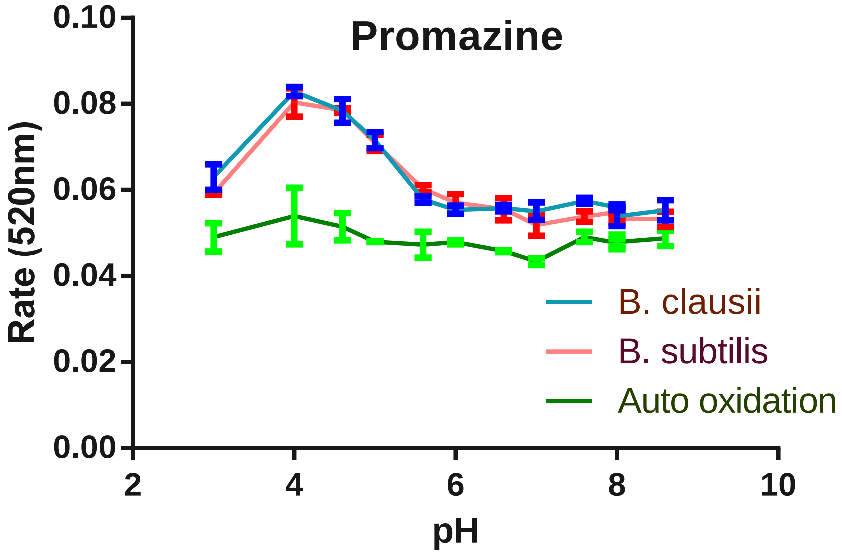
Promazine
Rate (520nm)
0.10
0.08
0.06
0.04
0.02
0.00
2
4
6
8
10
pH
B. clausii
B. subtilis
Auto oxidation

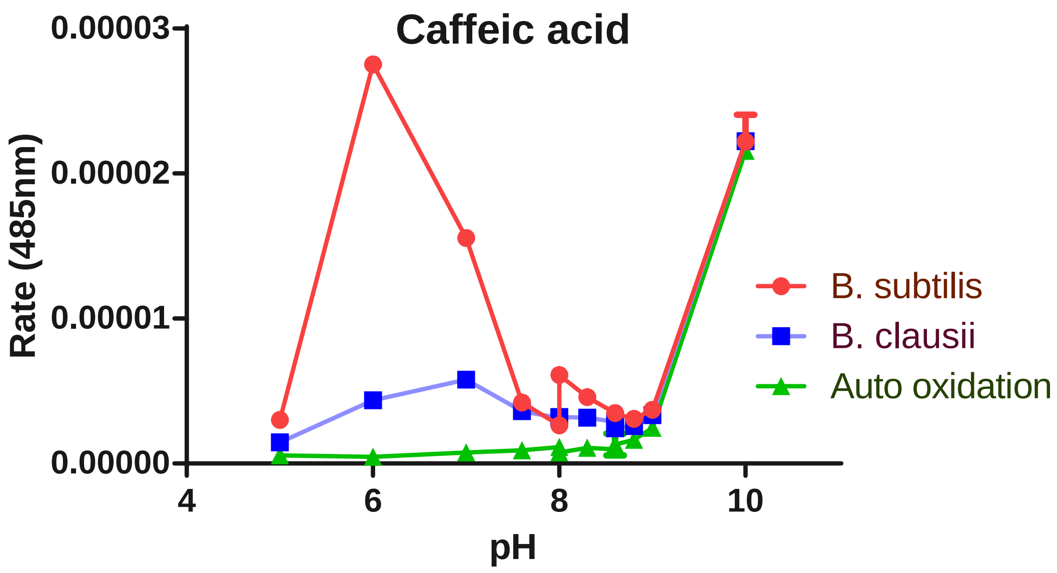
Caffeic acid
Rate (485nm)
0.00003
0.00002
0.00001
0.00000
4
6
8
10
pH
B. subtilis
B. clausii
Auto oxidation

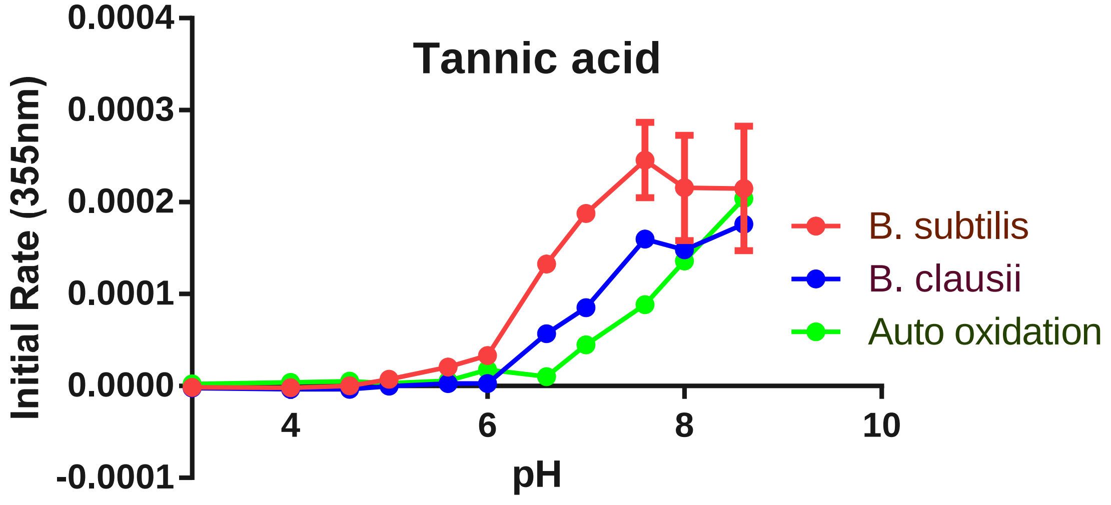

Tannic acid
Initial Rate (355nm)
0.0004
0.0003
0.0002
0.0001
0.0000
-0.0001
4
6
8
10
pH
B. subtilis
B. clausii
Auto oxidation

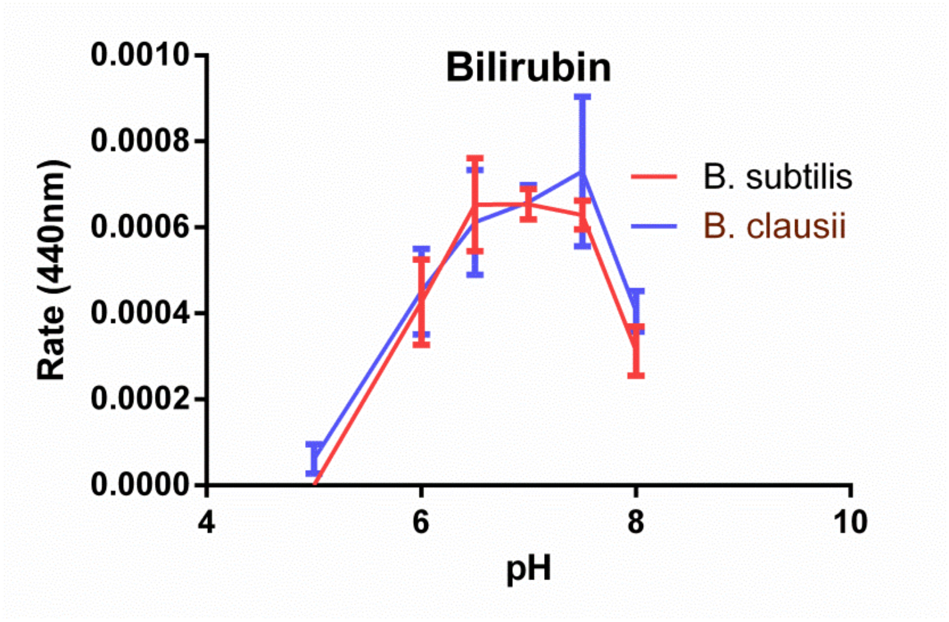

Bilirubin
Rate (440nm)
0.0010
0.0008
0.0006
0.0004
0.0002
0.0000
4
6
8
10
pH
B. subtilis
B. clausii

Figure S4. Residual activity of *B. subtilis* (dotted lines, squares) and *B. clausii* (solid lines, circles) cotA measured after 5 hours incubation at pH 3-10, 50°C.

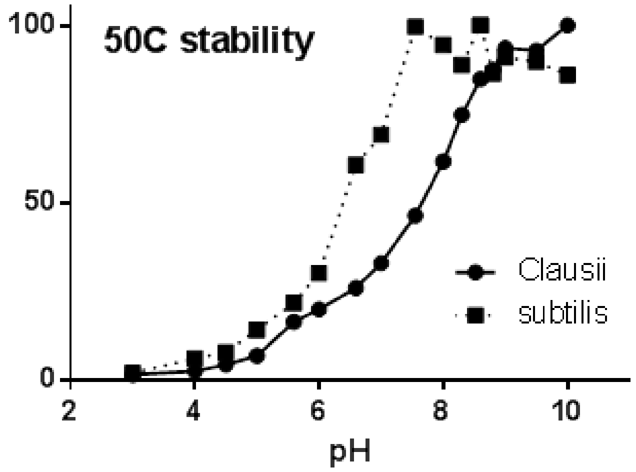

Figure S5: Residual activity of *B. subtilis* (dotted lines) and *B. clausii* (solid lines) cotA measured after 20 hours incubation at pH 5-10, 30°C. Data are in triplicate with the standard deviation error bars.

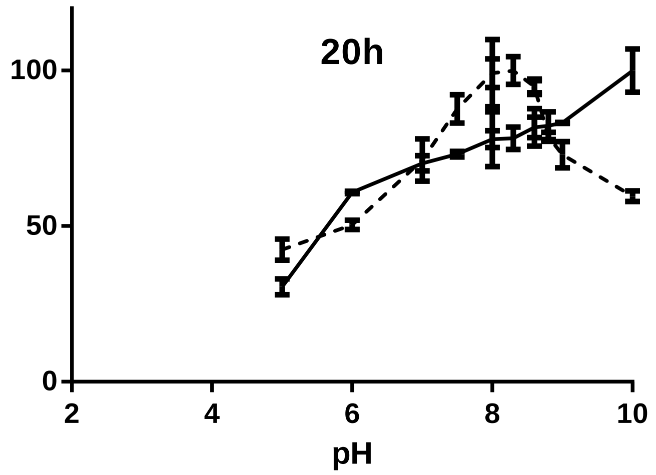

Figure S6: Michaelis-Menten analysis of *B. subtilis* cotA oxidation of DMP. Broken lines are fitted with non-competitive substrate inhibition, full lines without, giving similar k_{cat}/K_M.

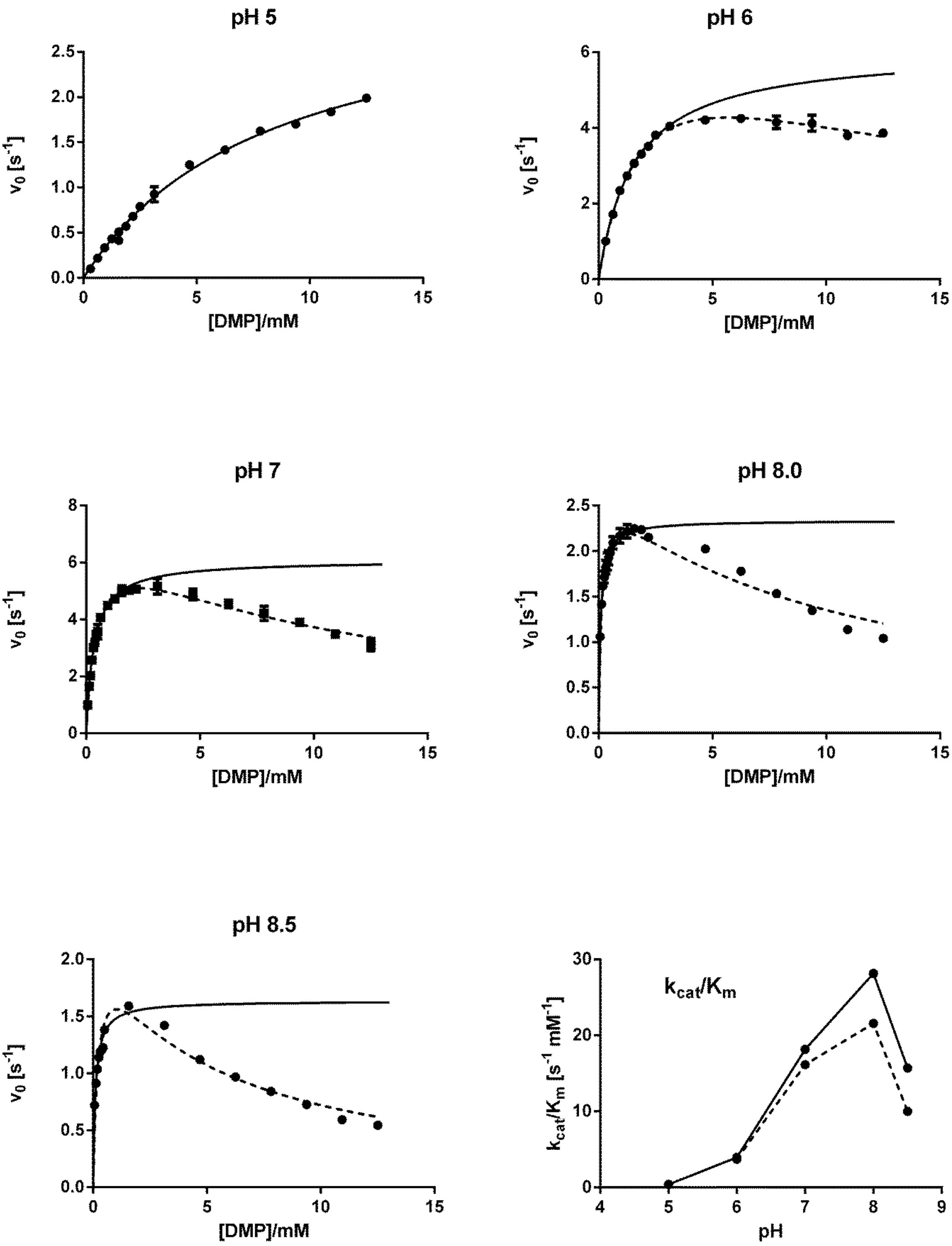

Figure S7: Michaelis-Menten analysis of *B. clausii* cotA oxidation of DMP. Broken lines are fitted with non-competitive substrate inhibition, full lines without, giving similar k_{cat}/K_M.

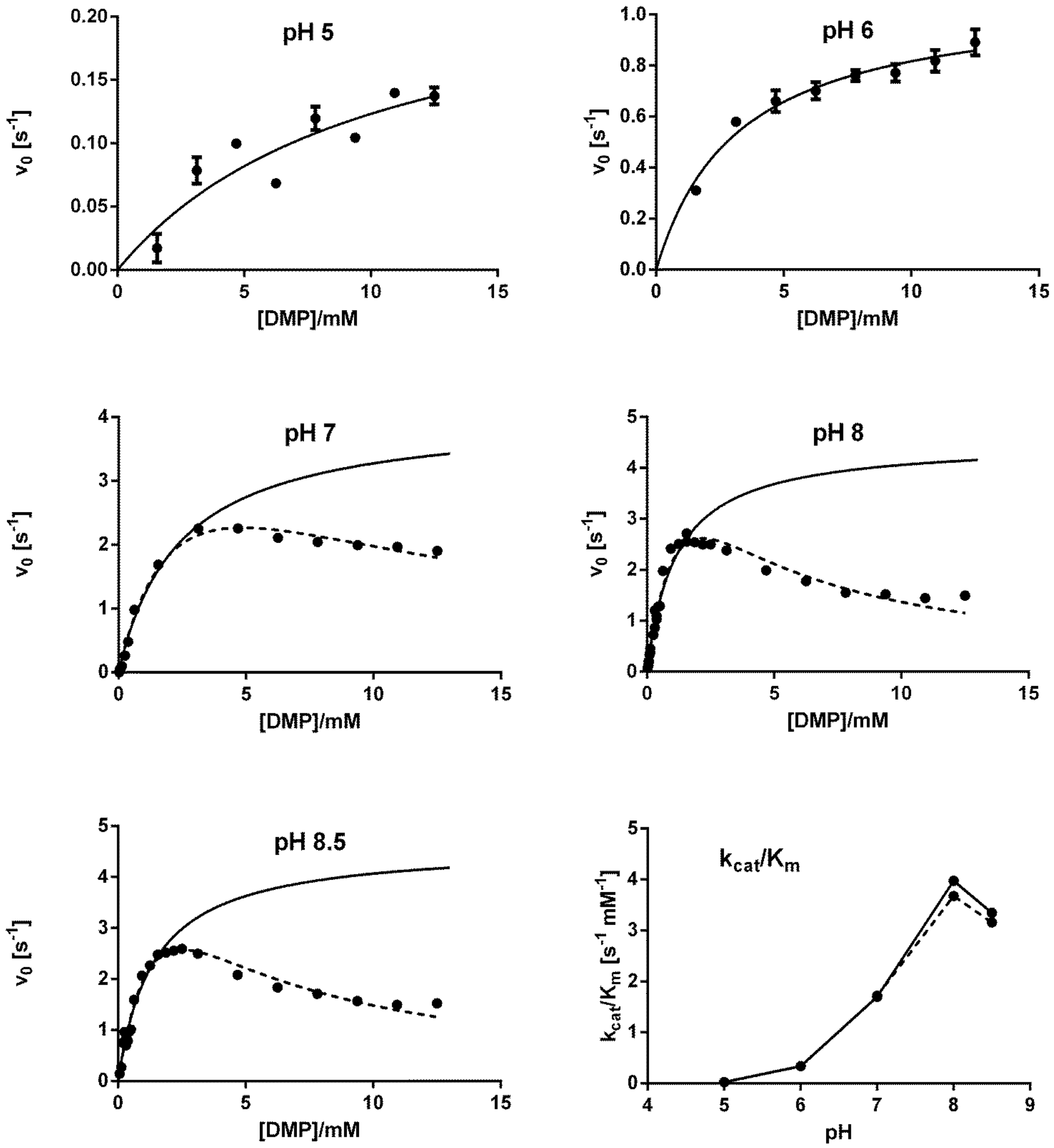

Figure S8: UV-VIS absorption profile of *B. clausii* cot A. A) Full spectrum with ABS_{280}=1.37 B) Zoom in on the 600nm feature with ABS_{280}=0.052

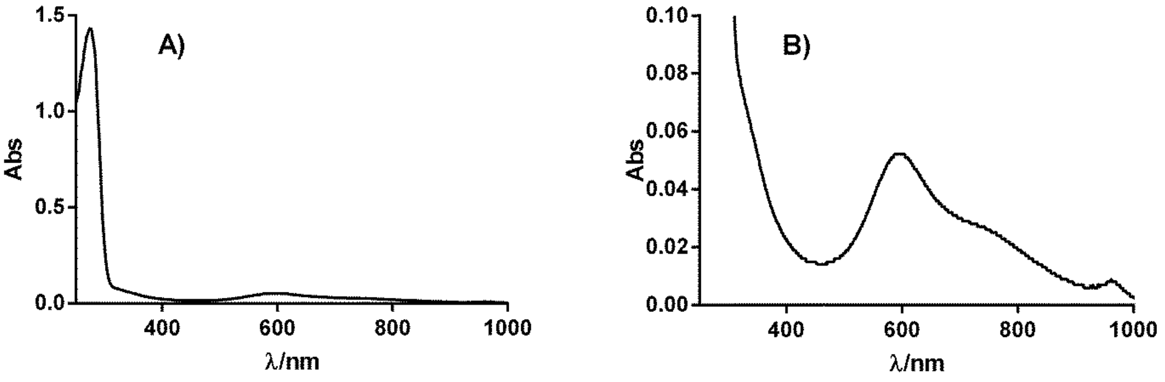

Supplement: File S1 — contains supporting data on sequence alignment of cotA orthologs (Figure S1), SDS page (Figure S2), activity profiles vs. pH without correcting for buffer differences and without subtraction of auto oxidation (Figure S3), data for pH-stability from activity measurements after incubation (Figure S4 and Figure S5), and data for the Michaelis Menten kinetics of cotAs from B. subtilis (Figure S6) and B. clausii (Figure S7). UV-VIS absorption profile of B. clausii cot A. (Figure S8). (PDF) [file pone.0099402.s001.pdf]
